# Vaccination against the Koala Retrovirus (KoRV): Problems and Strategies

**DOI:** 10.3390/ani11123555

**Published:** 2021-12-14

**Authors:** Joachim Denner

**Affiliations:** Institute of Virology, Free University Berlin, Robert von Ostertag-Str. 7-13, 14163 Berlin, Germany; Joachim.Denner@fu-berlin.de; Tel.: +49-(0)30-838-63059

**Keywords:** koala retrovirus, vaccination, neutralization, endogenous retroviruses, tolerance

## Abstract

**Simple Summary:**

The koala population is declining in northern Australia, and a major reason for this is the infection of the immunosuppressive koala retrovirus (KoRV), which is endogenous in many animals. This endogenous virus and its exogenous forms may induce lymphomas and immunodeficiency associated with opportunistic infections, including chlamydia infections. To generate a vaccine, we produced the recombinant surface and transmembrane envelope proteins of the KoRV and immunized goats, rats and mice. In all cases, we obtained antibodies which were able to neutralize the KoRV and recognize defined epitopes in the envelope proteins. However, we and others observed that koalas carrying the endogenous KoRV are tolerant, e.g., are unable to induce an immune response to the virus. Nevertheless, we propose that KoRV-negative animals will produce an antiviral immune response and will be protected when immunized with such a vaccine. This immunization will also reduce the number and severity of opportunistic infections because there is no KoRV-induced immunosuppression.

**Abstract:**

The koala retrovirus (KoRV) is spreading in the koala population from the north to the south of Australia and is also in the process of endogenization into the koala genome. Virus infection is associated with tumorigenesis and immunodeficiency and is contributing to the decline of the animal population. Antibody production is an excellent marker of retrovirus infection; however, animals carrying endogenous KoRV are tolerant. Therefore, the therapeutic immunization of animals carrying endogenous KoRV seems to be ineffective. Using the recombinant transmembrane (TM) envelope protein of the KoRV, we immunized goats, rats and mice, obtaining in all cases neutralizing antibodies which recognize epitopes in the fusion peptide proximal region (FPPR), and in the membrane-proximal external region (MPER). Immunizing several animal species with the corresponding TM envelope protein of the closely related porcine endogenous retrovirus (PERV), as well as the feline leukemia virus (FeLV), we also induced neutralizing antibodies with similar epitopes. Immunizing with the TM envelope protein in addition to the surface envelope proteins of all three viruses resulted in higher titers of neutralizing antibodies. Immunizing KoRV-negative koalas with our vaccine (which is composed of both envelope proteins) may protect these animals from infection, and these may be the starting points of a virus-free population.

## 1. Introduction

The koala (*Phascolarctos cinereus*) was recently listed under Australian government legislation as vulnerable in the northern states of Queensland, New South Wales and the Australian Capital Territory, but not in the southern states of Victoria and South Australia. Koala population decline is associated with habitat loss, hotter and extended droughts, dog attacks and vehicle collisions [1]. Besides these, diseases and infections, especially of the koala retrovirus (KoRV), are the main reason for the population decline.

The KoRV is a gammaretrovirus which can induce tumors and immunodeficiency. The most closely related gammaretroviruses to KoRV are the feline leukemia virus (FeLV), the murine leukemia viruses (MuLV), the porcine endogenous retrovirus (PERV), the *Melomys burtoni* retrovirus (MbRV) and the Gibbon ape leukemia virus (GaLV) [2,3]. MuLV, FeLV and GaLV induce leukemia and immunodeficiency in the infected hosts. Immunodefiency is usually associated with opportunistic infections [2]. Although the human immunodeficiency virus (HIV) and the feline immunodeficiency virus (FIV) are not closely related to the KoRV, the clinical picture of the immunodeficiency is quite similar. Among other features, HIV [4,5], FIV [6] and KoRV [7,8,9,10,11] infections are often associated with chlamydia infection. However, HIV-1 infections in humans are more often associated with *Pneumocystis jirovecii*, *Candida albicans*, and *Toxoplasma gondii* infections. It is unclear why chlamydia infections are the most common among KoRV-positive animals. One possibility could be that this is due to the high prevalence of these microorganisms in their habitat. Gammaretrovirus particles have been found in koalas with leukemia as early as 1988 [12]. Later, the virus was isolated from wild and zoo animals and was sequenced and characterized [13,14,15,16]. High plasma levels of KoRV were found in animals which developed leukemia and lymphoma, in addition to clinical chlamydiosis. In all cases, a clear correlation between virus load and severity of the disease was observed [17,18,19]. Furthermore, KoRV was found to be endogenous to many animals, i.e., it is integrated in the germ line of the koalas and is transmitted vertically in a Mendelian fashion [20]. The process of endogenization may have started less than 50,000 years ago [21] and is still ongoing in the southern population of koalas [22]. In addition to the endogenous KoRV (named KoRV-A), as well as some other, more ancient endogenous retroviral sequences found in the koala genome, exogenous KoRV-B (identical with KoRV-J) and eight other exogenous subtypes were identified, which obviously represent a recombination between KoRV-A and endogenous retroviral sequences [23]. KoRV-B uses a different receptor molecule to KoRV-A; KoRV-A uses the PiT1 receptor, a Na+-phosphate (Pi) cotransporter, while KoRV-B uses the thiamine transport protein 1 (THTR1) receptor. The promoter activity of the KoRV-B strain is stronger than that of KoRV-A, suggesting that KoRV-B may replicate more efficiently than KoRV-A [23]. KoRV-B seems to be responsible for the development of leukemia and lymphoma, as well as for immunosuppression, which is associated with opportunistic infections, whereas KoRV-A, based on its immunosuppressive properties, is mainly associated with opportunistic infections [18,24].

Most retroviruses, including HIV-1, FeLV and MuLV, are immunosuppressive viruses, and the immunosuppressive activity of KoRV was not only demonstrated by opportunistic infections, but also by alterations of the cytokine release in the infected animals. IL-6 expression was significantly greater in PBMCs from koalas infected with multiple subtypes than in those infected with KoRV-A alone [25]. KoRV-B infection is associated with up-regulation in IL-17A and IL-10 as well as in IFNγ, IL-6, IL-4 and TNFα [26]. It is important to note that in the case of FeLV, more cats die from opportunistic infections based on the virus-induced immunosuppression than from leukemia [2,27,28]. Immunosuppressive properties of the KoRV have been demonstrated in vitro [29].

Whereas in the case of the related PERV its origin from retroviral sequences in lesser Egyptian jerboas, rock hyraxes and murid species is well characterized [30], the origin of the KoRV is still unclear; however, it is possibly a transspecies transmission from rodents or bats [31,32].

## 2. Immunization Experiments with KoRV Envelope Proteins

**Figure 1:**
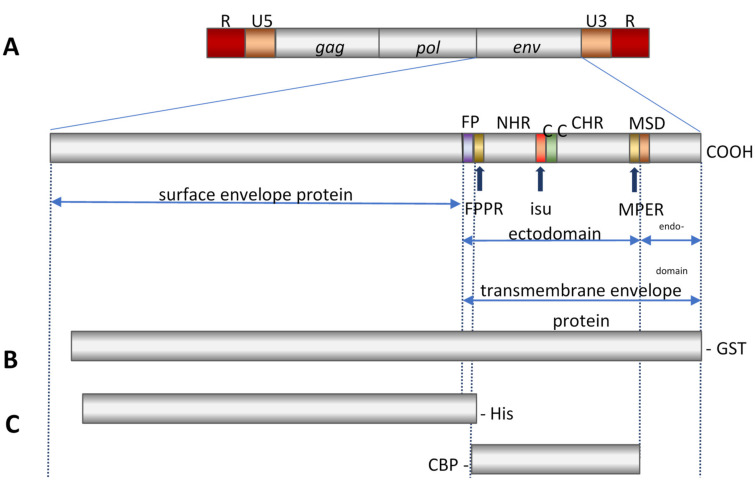
(**A**), Schematic presentation of the genome of the KoRV (R, repetitive sequence; U5, unique 5′ sequence; gag, group-specific antigen; pol, polymerase; env, envelope). In the transmembrane envelope protein, well characterized functional domains are indicated: FP, fusion peptide, FPPR, fusion peptide proximal region; NHR, N-terminal helical region; isu, immunosuppressive domain; C, cysteine; C-C, Cys-Cys loop; CHR, C-terminal helical region; MPER, membrane proximal external region; MSD, membrane spanning domain. (**B**), Recombinant precursor envelope protein fused to GST used by Olagoke et al. [33,34,35] for immunization, GST, glutathione S-transferase. (**C**), Recombinant proteins used by Fiebig et al. [36,37] for immunization, top: His-tag labeled surface envelope protein, bottom: ectodomain of the transmembrane envelope protein fused to CBP, CBP, calmodulin binding protein.

Since the envelope proteins of most viruses are the best target for neutralizing antibodies in order to prevent virus infections, we performed immunization experiments using recombinant envelope proteins of the KoRV. The SU envelope protein gp70 of KoRV binds to the receptor molecule, and the TM envelope protein, p15E, is responsible for the fusion between the virus and the cellular membranes. Both envelope proteins are produced in the infected cell as a precursor protein (gp85), which is then cleaved by a cellular protease (Figure 1). Antibodies that bind to certain parts of these proteins will neutralize the virus and inhibit infections.

In our immunization studies, we cloned and expressed the ectodomain of KoRV p15E [29] and the entire gp70 [36,37] in *E. coli*, purified and characterized the proteins, and used them alone or in combination for the immunization of rats and goats (Figure 1C). Neutralizing antibodies were determined in an assay based on the infection of human 293 cells with a KoRV. We used a virus isolated from a koala in a zoo in Düsseldorf, Germany [29,36], which is nearly identical to the KoRV isolated in Australia [13,19]. Neutralizing antibodies were also obtained when immunizing with DNA molecules encoding gp70 or the precursor protein gp85 [37].

**Figure 2:**
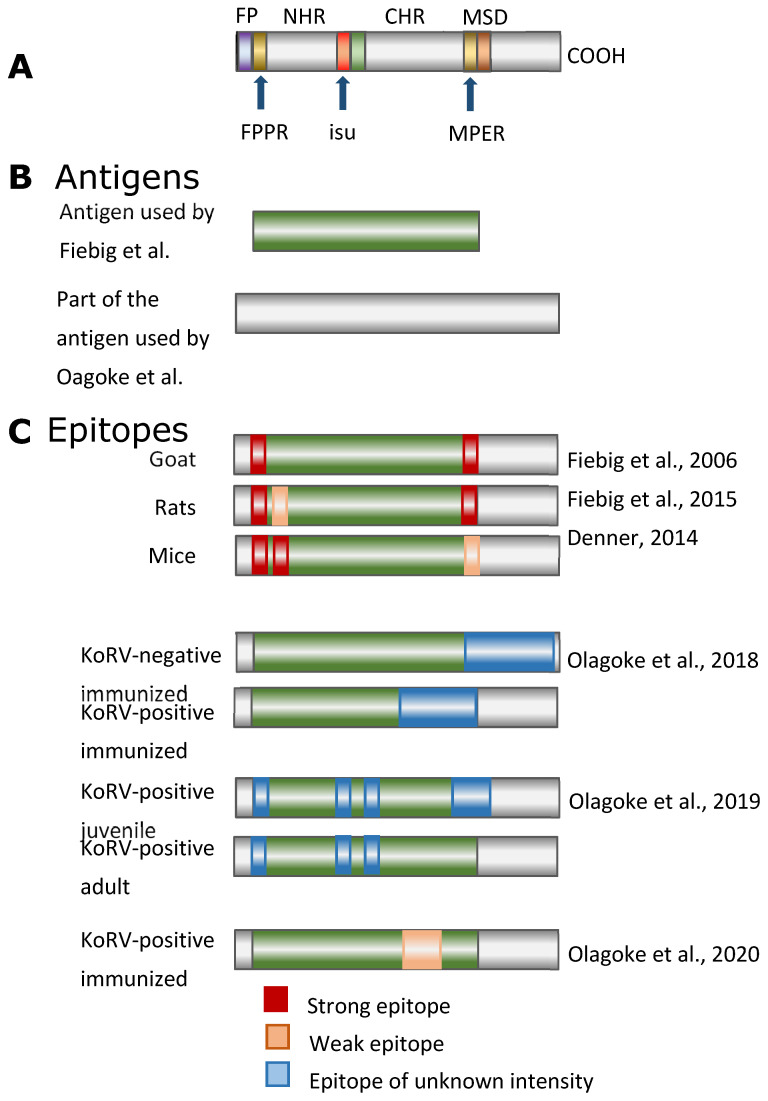
(**A**), Schematic presentation of the transmembrane envelope protein, the relevant domains are indicated (see Figure 1). (**B**), Antigens used for immunization. Top (green): antigen (ectodomain of the TM envelope protein) used by Fiebig et al. [29,36], reviewed in [37]; bottom (grey): antigen (part of the precursor envelope protein) used by Olagoke et al. [33,34,35]. (**C**), Localization of the epitopes recognized by antisera obtained after immunization with the above-mentioned antigens. The animals and the publications are indicated.

**Figure 3:**
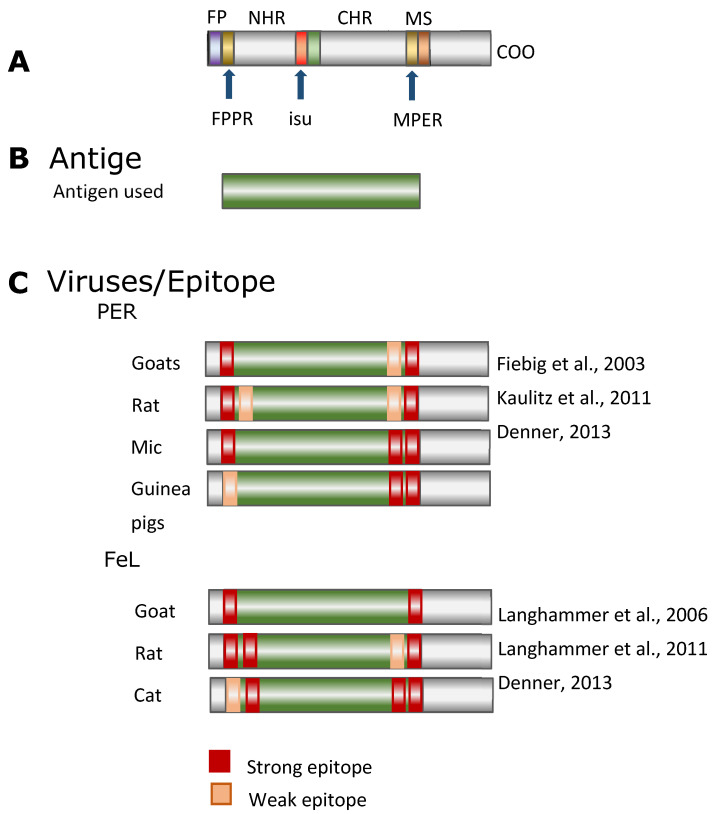
(**A**), Schematic presentation of the TM envelope proteins. The relevant domains are indicated (see Figure 1). (**B**), Antigen used for immunization: ectodomains of the related retroviruses PERV and FeLV, (**C**), localization of the epitopes recognized by antisera obtained after immunization with the ectodomains of TM envelope proteins of PERV and FeLV. The animals and the publications are indicated ([38,39,40,42,43,44]).

Using overlapping peptides corresponding to the p15E of KoRV, an epitope mapping of the immune sera was performed. Epitopes are the sequences in a protein, which are binding sites of the antibodies. Main epitopes were found in the fusion peptide proximal region (FPPR) of p15E and in the membrane proximal external region (MPER) near the membrane spanning domain (MSD), also called the transmembrane domain (Figure 2) [29,37,38]. Interestingly, the epitopes mapped in the case of different antisera against p15E of the KoRV were very similar in location and sequence to the epitopes identified when different animal species were immunized with p15E of the closely related PERVs [38,39,40,41] and FeLV [39,42,43,44] (Figure 2 and Figure 3). After immunizations against these three viruses, antibodies recognizing identical epitopes in the TM envelope protein were detected in all cases. This indicates that the epitope sequences represent parts of the protein which are crucial for infection, because interactions of the neutralizing antibodies with these sequences prevent infection.

**Figure 4:**
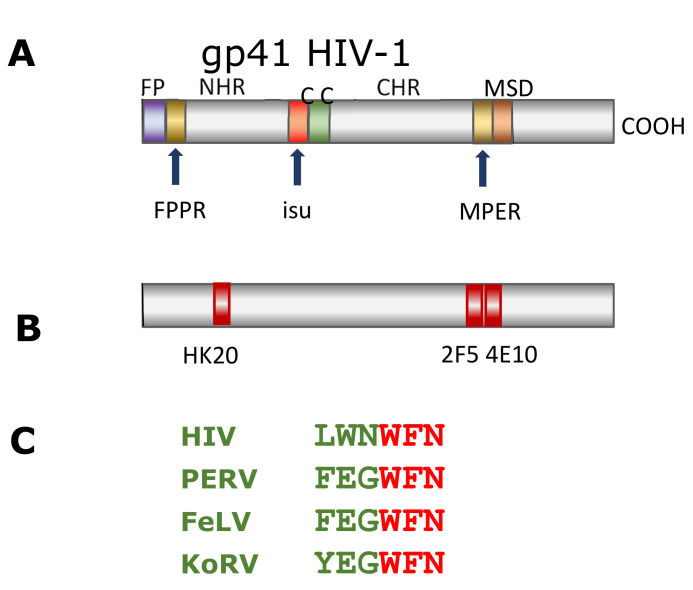
(**A**), Schematic presentation of the TM envelope protein gp41 of HIV-1. For abbreviations, see Figure 1. (**B**), Localization of the epitopes of broadly neutralizing monoclonal antibodies (HK20, 2F5 and 4E10) isolated from HIV-1 infected patients. (**C**), Sequence homology of the epitopes located in the MPER of the TM envelope proteins of different retroviruses, recognized by antibodies induced in species immunized with the TM envelope protein of PERV, FELV and KoRV. The epitope in the MPER of gp41 of HIV-1 is recognized by the monoclonal broadly neutralizing antibody 2F5, isolated from a HIV-1-infected individual.

In all cases (KoRV, FeLV and PERV), higher titers of neutralizing antibodies were achieved when the animals were immunized with a mixture of the TM and the SU envelope proteins [37,39,44,45]. Most interestingly, in the MPER of the TM envelope protein gp41 of HIV-1, a related epitope was found (Figure 4). Its corresponding antibody is broadly neutralizing, i.e., it neutralizes a high percentage of all HIV-1 clades [46], whereas in the case of the three gammaretroviruses (KoRV, FeLV and PERV), immunization with the linear TM envelope proteins induced successfully neutralizing antibodies using the linear TM envelope protein gp41 of HIV-1. We and others were unsuccessful in inducing antibodies neutralizing HIV-1 [39,46]. Even using trimerized gp41-derived molecules for immunization did not result in antibodies neutralizing HIV-1 [47]. It seems that in the case of HIV-1, the conformation of the TM envelope protein gp41, or its integration in the lipid membrane, is crucial for the induction of neutralizing antibodies, and that this is the reason why such antibodies could not be induced until now [39].

## 3. Screening of KoRV-Positive Animals for Neutralizing Antibodies

The antibody response is generally an important diagnostic tool for detecting retrovirus infections. However, when we analyzed koalas carrying the KoRV in their genome for antibodies against the virus, none of the koalas tested positive for virus-specific antibodies, suggesting a state of tolerance [48]. This was true for naturally infected animals from Australia carrying endogenous KoRV-A and seven animals from European zoos, two of which were also infected with KoRV-B. For Western blot analyses, lysates from purified viruses or recombinant envelope proteins were used, and additionally, we showed that the sera did not neutralize KoRV [48].

These data correlate well with our investigations on pigs. Pigs carry different but closely related endogenous retroviruses, such as PERV-A, PERV-B and PERV-C. When we screened pigs for antibodies against the envelope proteins of PERV, we found no antibodies [49]. Furthermore, we immunized pigs with the TM protein p15E, and the recombinant SU protein gp70/rp52 produced in *E. coli*. Additionally, no antibodies were detected [49,50], indicating that pigs are tolerant against the envelope protein and suggesting that these antigens are expressed in early ontogenesis and recognized as “self”.

## 4. Immunization Experiments In Vivo

Despite these results, which suggest that the immunization of animals which carry and express endogenous KoRV-A may be not successful, Waugh et al. [51] reported that antibodies against KoRV in KoRV-infected animals are using the TM and SU envelope proteins prepared in our laboratory (see C in Figure 1). Olagoke et al. [33,34] performed the first immunization studies. These authors reported an increase in circulating anti-KoRV IgG levels. However, the antibody titers were very low, and it was unclear whether the authors had discriminated between the antibodies and the envelope or between the GST part of the fusion protein used for immunization and the part used in the ELISA for antibody detection (Figure 1B). In contrast to Western blot analyses, ELISAs have the disadvantage that in the case of the antigen, which was produced in bacteria, they will not be fully purified; antibodies in the koala-recognizing bacterial antigens may interact with the bacterial contamination in the protein preparation used as an antigen to give false positive results. ELISAs are quick to perform, and one advantage is the possibility to detect antibodies against intramolecular conformational epitopes. Western blot analyses are more time consuming, and they cannot, due to the sodium dodecyl sulfate polyacrylamide gel electrophoresis (SDS-PAGE), recognize such epitopes.

In addition to the ELISA, the authors performed a neutralization assay based on the detection of integrated KoRV provirus in Hep2 cells. The sera from immunized koalas showed a reduction in the virus infection in the assays; however, the authors did not demonstrate that this was due to antibodies, nor did they test sera from animals immunized solely with the adjuvant. Antiviral factors may have been induced by immunization with the adjuvant, which may act in the neutralization assay. Such antiviral factors produced by CD8^+^ T cells have been described in the case of HIV infections [52]. In addition, intracellular restriction factors may have been activated in the process of immunization [53]. These CD8^+^ T cell factors and restriction factors may have also played a role in the observed reduction in the virus load in the immunized animals [33,34,35].

In addition, the authors identified the epitopes recognized by the antibodies after immunization using overlapping peptides corresponding to the envelope protein used for immunization (Figure 2). The epitopes they described differ significantly from the epitopes detected in our immunization experiments. We think that the epitopes detected in our experiments are relevant for virus neutralization, since these were found after the immunization of certein species (mice, rat, goat) with the TM envelope proteins of PERV, FeLV and KoRV, as well as in HIV-1-infected individuals (Figure 2 and Figure 4). Therefore, it cannot be excluded that the authors measured unspecific antibodies, especially because the authors did not determine the epitopes of the sera before immunization [35]. In a separate study, they analyzed epitopes from sera of KoRV-positive juvenile and adult animals at different time points of their lives. These sera from some of the animals detected epitopes in FPPR, some in the immunosuppressive domain, and some in the Cys-Cys-loop (C-C loop) in the TM envelope protein (see Figure 1). Only the juvenile animals at 4.5 years of age, but not at 1 year of age, recognized epitopes in the MPER. Sera from older animals did not react against epitopes in the MPER [34]. The antibodies against the immunosuppressive domain and against the Cys-Cys-loop are of interest, since such antibodies were also found in HIV-1-infected individuals, and the loop is an immunodominant epitope in HIV-1-infected individuals. Nearly all patients produce antibodies against this loop [54,55]. However, it is an immunodominant epitope, because uninfected individuals also have cross-reacting antibodies. When the authors compared non-vaccinated animals with vaccinated animals (both of which were positive for endogenous KoRV-A), an increase in the frequency of binding to epitopes corresponding to the MPER and to the C-terminal helix and a decrease in the frequency of binding to epitopes corresponding to the FPPR was observed [34]. In older koalas, no antibodies against the MPER were detected [34]. In a later study [35], high antibody binding was observed against the SU envelope protein, and in the case of the TM envelope protein, significant antibody binding was only observed against peptides corresponding to the MPER in KoRV-positive animals. However, all these antibodies were found already in Week 0, i.e., before the vaccine could induce antibodies. New antibodies appeared only against a region in the C-terminal end of the SU envelope protein and in the cytoplasmic domain (endodomain) of p15E, which is inside the virus particle.

It will be important to find the reasons for the different data concerning the immune response of koalas against the envelope proteins of KoRV. There are data showing that animals carrying the endogenous KoRV are tolerant and thus unable to produce antibodies [48]. On the other hand, there are data showing that such animals produce neutralizing antibodies [33,34,35]. The first data set was recently confirmed by Joyce et al. [56]. They could also not find antibodies against the envelope protein in non-immunized animals carrying the endogenous KoRV. For the tests, they used the TM envelope protein as a trimer, which was well characterized by electrophoresis and electron microscopy. Two independent methods, ELISA and Western blot analysis, demonstrated the absence of antibodies against the envelope protein of the animals carrying the endogenous KoRV [56].

## 5. Conclusions and Outlook: How to Save Koalas by Vaccination against the KoRV

Our study [48] and the study conducted by Joyce et al. [56] both indicate that koalas which have the endogenous KoRV in their genome do not produce antibodies against the virus because they are tolerant. This suggests that additional immunization will be unable to produce binding and neutralizing antibodies able to reduce the virus load of these animals. This is supported by the finding that therapeutic vaccination was also inefficient in clinically healthy cats persistently infected with FeLV [57].

The TM envelope protein, p15E of KoRV and the TM envelope protein of other gammaretroviruses are suitable to be used as antigens to induce neutralizing antibodies. The KoRV TM envelope protein is highly conserved among koalas from different geographic regions, and several analyses suggested that p15E was under the purifying selection [58]. Important epitopes and domains were highly conserved across the p15E sequences in all reported KoRVs. These results support the potential use of p15E, together with the SU envelope protein for KoRV vaccine development.

It will be important to investigate whether the recombinant ectodomain of the TM and the SU envelope proteins of the KoRV, which successfully induced neutralizing antibodies in different species (goat, rat and mouse) (Figure 2), will also induce such neutralizing antibodies in koalas which do not carry the endogenous KoRV in their genome. Since antibodies against the FeLV, produced following the same principle, were able to protect cats from FeLV-induced leukemia, immunization with the TM and the SU envelope proteins of the KoRV may be protective in koalas not carrying the endogenous KoRV in their genome. Starting with an immunized and protected koala colony, vaccinated animals can later be released into the wild. Preventing KoRV infection will also reduce the number and severity of opportunistic infections, including chlamydia infections, because there is no KoRV-induced immunosuppression. The antiretroviral treatment of HIV-1-infected individuals significantly reduces the virus load and the immunosuppression, and this also significantly reduces the opportunistic infections [59].

Since there is the risk that the whole koala population will become infected soon, immediate action is required. According to Stephenson et al. [60], 41% of the animals were KoRV positive, 57% were KoRV negative and 2% were inconclusive in the southern koala populations (from Victoria and South Australia). In Queensland, 100% of the animals are infected [61]. It is assumed that the infection started at the north coast, but it is difficult to estimate how fast the virus infection is spreading in Australia. While there are indications that the virus was ubiquitous in Queensland koalas in the late 19th century [61], the process of endogenization may have started less than 50,000 years ago [60,61]. As long as there are still KoRV-negative and genetically healthy animals without endogenous KoRV in their genome, the population can be saved by vaccination.

Whether it will be possible to immunize koalas carrying the endogenous KoRV-A provirus in their genome against the very short, but different receptor-binding domain in the SU envelope protein of KoRV-B and other non-KoRV-A, raises an interesting question to be answered in the future. Since vaccination against the SARS-CoV-2 using mRNA vaccines is proving successful [62], it could be suggested that this strategy is also of use when immunizing against the KoRV.

## Data Availability

Not applicable.
